# High efavirenz levels but not neurofilament light plasma levels are associated with poor neurocognitive functioning in asymptomatic HIV patients

**DOI:** 10.1007/s13365-020-00860-1

**Published:** 2020-06-10

**Authors:** Charlotte S. Hakkers, Anne Marie Hermans, Erik M. van Maarseveen, Charlotte E. Teunissen, Inge M. W. Verberk, Joop E. Arends, Andy I. M. Hoepelman

**Affiliations:** 1 Department of Internal Medicine, section Infectious Diseases, University Medical Center (UMC) Utrecht, Utrecht University, PO Box 85500, 3508 GA Utrecht, the Netherlands; 2 Division of Laboratory and Pharmacy, Clinical Pharmacy, University Medical Center (UMC) Utrecht, Utrecht, the Netherlands; 3 Department of Clinical Chemistry, Amsterdam Neuroscience Neurochemistry laboratory, Vrije Universiteit Amsterdam, Amsterdam UMC, Amsterdam, the Netherlands

**Keywords:** HIV, Efavirenz, Cognition, Asymptomatic, Neurofilament light

## Abstract

The aim of this study is to assess the effect of efavirenz exposure on neurocognitive functioning and investigate plasma neurofilament light (Nfl) as a biomarker for neurocognitive damage. Sub-analysis of the ESCAPE-study, a randomised controlled trial where virologically suppressed, cognitively asymptomatic HIV patients were randomised (2:1) to switch to rilpivirine or continue on efavirenz. At baseline and week 12, patients underwent an extensive neuropsychological assessment (NPA), and serum efavirenz concentration and plasma Nfl levels were measured. Subgroups of elevated (≥ 4.0 mg/L) and therapeutic (0.74 to< 4.0 mg/L) baseline efavirenz concentration were made. Differences between these groups in baseline NPA Z-scores and in delta scores after efavirenz discontinuation were assessed. Nfl level was measured using an ELISA analysis using single molecule array (Simoa) technology. Correlation of plasma NFL with NPA Z-scores was evaluated using a linear mixed model. The elevated group consisted of 6 patients and the therapeutic group of 48. At baseline, the elevated group showed lower composite Z-scores (median − 1.03; IQR 0.87 versus 0.27; 0.79. *p* 0.02). This effect was also seen on the subdomains verbal (*p* 0.01), executive functioning (*p* 0.02), attention (*p* < 0.01) and speed (*p* 0.01). In the switch group, the elevated group improved more on composite scores after discontinuing efavirenz (mean 0.58; SD 0.32 versus 0.22; 0.54, *p* 0.15). No association between plasma Nfl and composite Z-score was found. High efavirenz exposure is associated with worse cognitive functioning compared with patients with therapeutic concentrations. Plasma Nfl is not a suitable biomarker to measure cognitive damage in this group.

**Electronic supplementary material:**

The online version of this article (10.1007/s13365-020-00860-1) contains supplementary material, which is available to authorized users.

## Introduction

Antiretroviral agents used to treat infection with human immunodeficiency virus 1 (HIV) have been associated with neurocognitive Impairment (NCI) (Robertson et al. 2010; Shah et al. 2016). Especially efavirenz, a non-nucleoside reverse transcriptase inhibitor, is known for its neurological and psychiatric side effects and has been associated with higher rates of NCI (Muñoz-Moreno et al. 2009; Arendt et al. 2007; Ciccarelli et al. 2011; Decloedt and Maartens 2013; Abers et al. 2014; Gaida et al. 2016). Even in patients without clinically manifested cognitive complaints (cognitively asymptomatic patients), a negative effect of efavirenz on cognition has been shown (Robertson et al. 2010). In the ESCAPE study, we found that discontinuing efavirenz led to an objective improvement in neurocognitive functioning in a group of asymptomatic people with HIV (Hakkers et al. 2019). However, efavirenz remains a popular choice in antiretroviral therapy, mainly in resource-limited settings, mainly because is it part of Atripla, a single-tablet regime that is relatively cheap and has a convenient once a day dosage.

Multiple mechanisms on how efavirenz causes neurotoxicity have been described through in vitro and in vivo studies. For instance, a neurotoxic effect of efavirenz and its major metabolite 8-hydroxy-efavirenz was found in neuronal cultures, affecting dendrites and dendritic processes (Robertson et al. 2012; Tovar-y-Romo et al. 2012; Ciavatta et al. 2017). Moreover, studies have shown a detrimental effect of efavirenz on the blood-brain barrier and on neuronal action potential thresholds (Bertrand and Toborek 2015; Ciavatta et al. 2017). Effects on other mechanisms such as calcium homoeostasis or creatine kinase metabolism have also been shown (Streck et al. 2008; Tovar-y-Romo et al. 2012). Most studies show a concentration-dependent effect of efavirenz on CNS side effects such as impaired concentration, and this is most notable from serum concentrations above > 4 mg/L (Marzolini et al. 2001; Borand et al. 2014). It might therefore be interesting to investigate the effect of high serum efavirenz concentrations on cognition.

Furthermore, there is a need for fast and patient-friendly diagnostic tools (biomarkers) for diagnosing neurocognitive damage, because the current gold standard, a neuropsychological assessment (NPA), is timely and expensive. Recent interest has emerged in a protein called neurofilament light (Nfl) which is a major structural component of axons and is released into the cerebrospinal fluid (CSF) and blood upon axonal damage and neuronal death (Varhaug et al. 2018). CSF Nfl is elevated in patients suffering from HIV-associated dementia (Yilmaz et al. 2017). The recent development of an ultrasensitive immunoassay for plasma Nfl using single molecule array (Simoa) technology allows testing for neurocognitive injury in plasma instead of CSF (Kuhle et al. 2016). Several studies have established that there is a strong correlation between plasma Nfl and CSF Nfl (Rojas et al. 2016; Meeter et al. 2016; Wilke et al. 2016; Piehl et al. 2017; Kovacs et al. 2017). Besides HIV infection, plasma Nfl has been investigated in neurological conditions such as frontotemporal dementia, multiple sclerosis and Creutzfeldt disease and proven to be useful as a biomarker of neurodegeneration (Rohrer et al. 2016; Steinacker et al. 2016; Kuhle et al. 2017). The results from two studies suggest that plasma Nfl may provide an almost equally good indicator of active CNS injury compared with CSF Nfl in people with HIV (Gisslén et al. 2016; Anderson et al. 2018).

Given the demonstrated improvement in neurocognitive functioning in earlier studies after discontinuing efavirenz, the main hypothesis of this study is that this observed effect is related to efavirenz exposure, measured by elevated serum drug levels. Furthermore, we investigate the hypothesis that a high exposure to efavirenz leads to axonal damage and/or neuronal cell death, which can be measured by plasma Nfl.

## Methods

### Participants

This study is a sub-analysis of the ESCAPE trial (Effect of SwitChing AtriPla to Eviplera on neurocognitive and emotional functioning) which was previously published (Hakkers et al. 2019). In short, this randomised controlled trial included neurologically asymptomatic, stable (i.e. undetectable viral load), HIV-infected male patients on efavirenz/emtricitabine/tenofovir (Atripla) for at least 6 months, aged from 25 to 50 years old. Participants were excluded if they had active or past CNS opportunistic infections, active psychiatric of neurologic disorders and/or a history or evidence of alcohol or drug abuse. The study was performed according to the declaration of Helsinki and was reviewed and approved by the medical ethical board of the University Medical Center Utrecht. All participants signed written informed consent (Hakkers et al. 2019).

### Study design

In the ESCAPE study, participants were randomised to the switch group, where they would switch to rilpivirine/emtricitabine/tenofovir (Eviplera), or the control group (continuing on Atripla) with a randomisation ratio of 2:1. At baseline and study week 12, blood was collected for serum measurement of efavirenz concentration and plasma measurement of NFL concentrations, as well as HIV-RNA and CD4 cell count. Also, a comprehensive NPA was performed. Seven cognitive domains were tested by the NPA: language, learning and memory, executive functioning, attention/working memory, speed of information processing and psychomotor speed (Hakkers et al. 2019). The different subtests used were as follows: Controlled Oral Word Association Test (Schmand et al. 2008); category fluency(Van der Elst et al. 2006); Rey Auditory Verbal Learning Test; Rey complex figure test (A Rey 1941); trail making test part A and B (Reitan and Wolfson 1985); Brixton Spatial Anticipation Test (Burgess and Shallice 1997); visual elevator (Robertson et al. 1994); Paced Auditory Serial Addition Test (Gronwall and Samspon 1974); Letter-Number-Sequencing WAIS-IV NL, Digit Symbol WAIS-IV NL and Symbol Search WAIS-IV NL (Wechsler 2013); and Grooved Pegboard (dominant and non-dominant)(Roy and Square-Storer 1994). When possible, different test versions were used on baseline and for week 12, in order to minimise repeated testing effects. By using Dutch norm data, domain Z-scores were calculated, and a composite Z-score was calculated taking all different domains into account. A Z-score correlates to the amount of standard deviations a person deviates from the mean of the norm group, so a higher Z-score means a better performance.

### Efavirenz concentration analysis

For the analysis of efavirenz serum concentrations, an aliquot of 50 μL serum was diluted with 200 μL 0.1 M zinc sulphate and 500 μL internal standard solution. The vials were vortexed for 1 min and centrifuged at 13,000 rpm for 5 min, and 25 μL was injected on the LC-MS/MS system, a Thermo Fisher Scientific (Waltham, MA, USA) triple quadrupole Quantum Access LC–MS/MS system with a Surveyor MS pump and a Surveyor Plus autosampler with an integrated column oven. The Quantum Access mass selective detector was set in electrospray positive ionisation mode and performed selected reaction monitoring. Data acquisition and data processing were performed using Xcalibur software version 2.10. The analytical column was a HyPurity C18 50 mm × 2.1 mm column with 3 μm particle size (Thermo Scientific). Analytes were detected by a Thermo Fisher Scientific (Waltham, MA) triple quadrupole Quantum Access detector using heated electrospray ionisation (HESI). Ions monitored in the selected reaction monitoring (SRM) mode regression coefficient (*R*^2^) were 0.98. The lower limit of quantification (LLQ) was 0.1 mg/L. Accuracy and precision were within the maximum tolerated bias and coefficient of variation, 20% for LLQ and 15% for medium and high quality controls.

### Plasma NFL analysis

Plasma NfL concentrations were quantified in blood by the Neurochemistry Laboratory, Amsterdam UMC, location VUmc, using an in-house developed Homebrew Simoa assay, validated according to the standardised international protocols and described in detail elsewhere (Limberg et al. 2015; Kuhle et al. 2016). The monoclonal NfL capture antibody (Anti NfL mAb 47:3; UmanDiagnostics, Umeå, Sweden) was titrated to 0.3 mg/mL and chemically coupled to paramagnetic carboxylated beads (Quanterix, Lexington, USA). The assay had a lower limit of quantification of 1.54 pg/mL. All samples were measured in duplicate.

### Statistical analysis

Initially, all participants were divided into two groups: elevated baseline concentration of efavirenz, meaning a concentration of ≥ 4.0 mg/L, and therapeutic baseline concentration of efavirenz, meaning a concentration of < 4.0 mg/L. This division was made at baseline in the entire population to assess concentration effects on NPA Z-score in a cross-sectional manner and again at baseline in only the switch group to investigate longitudinal effects.

Subsequently, differences in baseline characteristics (age, education, employment status, BMI, duration of HIV infection and cART, current and nadir CD4) between elevated and therapeutic concentration groups were investigated. For categorical variables, either a chi-squared test or Fisher’s exact test (if values were expected to be below five) was used, and for continuous variables, either an independent sample *t* test for normal distribution or a Mann-Whitney *U* test for skewed distribution was used. Level of education according to the Verhage scale (Verhage 1964) was divided in high (group six and seven) and low (< group six). Differences between NPA Z-scores of the two groups at baseline were analysed using an independent samples *t* test for normal distribution or a Mann-Whitney *U* test for skewed distribution. With the same approach, difference (‘delta’) scores of NPA Z-scores were assessed in the switch group.

Then, to evaluate the effect of plasma NFL on composite Z-score, a linear mixed model with random intercept was built combining measurements at baseline and end of study, resulting in 108 measurements. The variable concentration of efavirenz was transformed to a log variable to ensure better fitting of the model. A restricted maximum likelihood linear mixed model was run to investigate the effect of plasma NfL on composite Z-score, including the following covariates (i.e. fixed effects): age, neuropsychiatric comedication, months on cART, CD4 count, HIV disease duration, CD4 nadir, concentration of efavirenz and ‘time point in study’ (marked as a categorical variable). Duration of efavirenz use in terms of months was left out of the analysis because of the by now reached steady state of this drug. Participants were defined as random effects to correct for multiple measurements in one participant. After building a full mixed model, the backward method according to the principle of a linear regression was used to investigate factors of interest (i.e. plasma NfL and other fixed effects) by building a final model. This model was again fit by restricted maximum likelihood.

Finally, to investigate disturbing influences on plasma NfL alone, a univariable and multivariable linear regression was performed. Factors with a *p* value < 0.20 in the univariable models, or with a scientific rationale, were entered in the multivariable linear regression model. The backward method was used, and *p* < 0.05 was applied as a cut-off level for acceptance. Only five variables were entered, approximately one per every ten participants.

Mixed model analyses were performed using R Statistical Software version 3.3.2; for the remaining analyses, IBM SPSS version 21 was used. Overall, an alpha of < 0.05 was used as a cut-off.

## Results

**Fig. 1:**
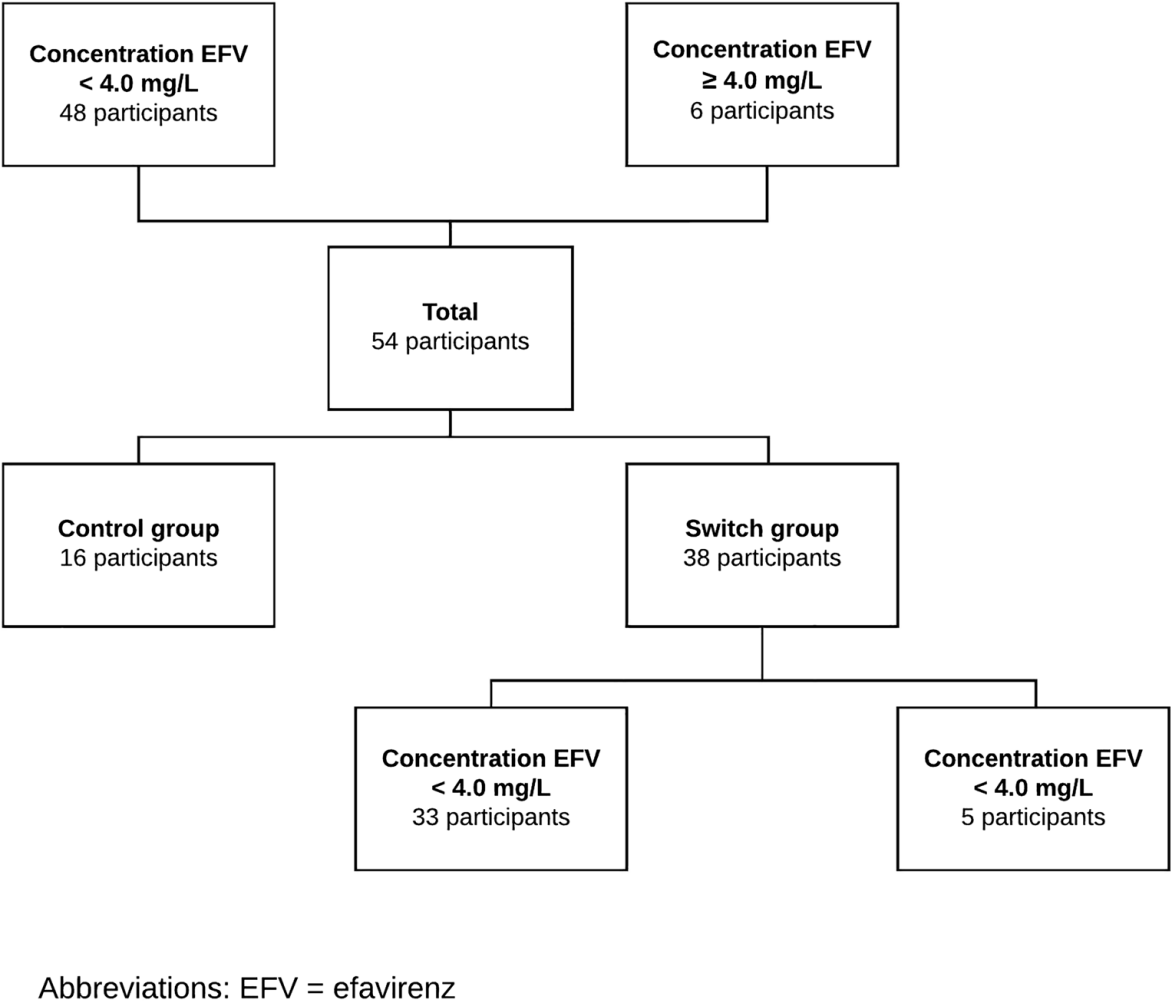
Flowchart. EFV, efavirenz

In the ESCAPE trial, a total of 54 participants were included. Participants were divided into therapeutic baseline efavirenz concentration (*n* = 48) and elevated baseline efavirenz concentration (*n* = 6) (Fig. 1). There was no significant difference between patient characteristics of these groups at baseline, except for employment status (*p* = 0.03) (Table 1).

**Table 1:** Baseline characteristics

Variable	Total *N* = 54 (100%)	Concentration EFV < 4.0 ng/mL *N* = 48 (88.9%)	Concentration EFV ≥ 4.0 ng/mL *N* = 6 (11.1%)	*p* value
Age, in years	41 (11)	41 (11)	43 (11)	0.81
High educational attainment according to Verhage (group 6 and 7), *N* (%)	26 (48.1)	23 (47.9)	3 (50.0)	0.63
Employed *N* (%)	51 (94.4)	47 (97.9)	4 (66.7)	0.03
BMI, in kg/m^2^ mean ± SD	24.1 ± 3.3	24.1 ± 3.5	23.4 ± 2.7	0.52
Use of neuropsychiatric medication, *N* (%)	7 (13.0)	6 (12.5)	1 (16.7)	0.58
cART, in months	58 (51)	58 (50)	58 (85)	0.99
EFV treatment duration, in months mean ± SD	56 ± 28	56 ± 29	56 ± 24	0.88
CD4 count, in cell/mm3	605 (268)	620 (255)	546 (365)	0.46
CD4 nadir, in cell/mm^3^	295 (138)	275 (144)	355 (100)	0.05
HIV disease duration, in months	92 (56)	92 (51)	85 (140)	0.85
EFV concentration, in mg/L	2.16 (1.50)	1.80 (1.31)	7.11 (9.67)	<0.01
Plasma NFL, in pg/mL	21.6 (16.6)	21.6 (16.8)	20.7 (24.3)	0.82

Values shown as median (IQR), unless otherwise specified

*Difference considered significant (*p* value < 0.05)

*N* number, *EFV* efavirenz, *IQR* interquartile range, *BMI* body mass index, *SD* standard deviation, *cART* combination antiretroviral therapy, *HIV* human immunodeficiency virus, *NFL* neurofilament light

### Effect of efavirenz concentration on NPA

Participants with an elevated concentration of efavirenz at baseline had a significantly lower NPA composite Z-score (i.e. decreased cognitive function) at baseline compared with those with a therapeutic efavirenz concentration (− 1.03; IQR 0.87 versus 0.27; IQR 0.79, *p* = 0.02). When analysing the specific domains, elevated efavirenz concentrations were associated with lower NPA scores in the following domains: verbal (− 0.66; SD 0.83 versus 0.41; SD 0.93, *p* = 0.01), executive functioning (− 0.59; SD 0.78 versus 0.26; SD 0.83, *p* = 0.02), attention (− 2.05; SD 1.33 versus − 0.35; SD 1.04, *p* < 0.01) and speed (− 1.08; SD 0.81 versus 0.07; SD 1.00, *p* = 0.01) (Table 2).

**Table 2:** NPA Z-scores at baseline in normal and high concentration of EFV groups

Type of NPA Z-score	Concentration EFV < 4.0 mg/L	Concentration EFV ≥ 4.0 mg/L	*p* value
Composite median (IQR)	0.27 (0.79)	− 1.03 (0.87)	0.02
Domain verbal	0.41 ± 0.93	− 0.66 ± 0.83	0.01
Domain memory	− 0.04 ± 0.50	− 0.18 ± 0.46	0.52
Domain executive functioning	0.26 ± 0.83	− 0.59 ± 0.78	0.02
Domain attention	− 0.35 ± 1.04	− 2.05 ± 1.33	< 0.01
Domain speed	0.07 ± 1.00	− 1.08 ± 0.81	0.01
Domain motor median (IQR)	0.18 (1.24)	− 1.14 (2.98)	0.08
Domain learning	0.33 ± 0.68	0.12 ± 1.02	0.52

**Fig. 2:**
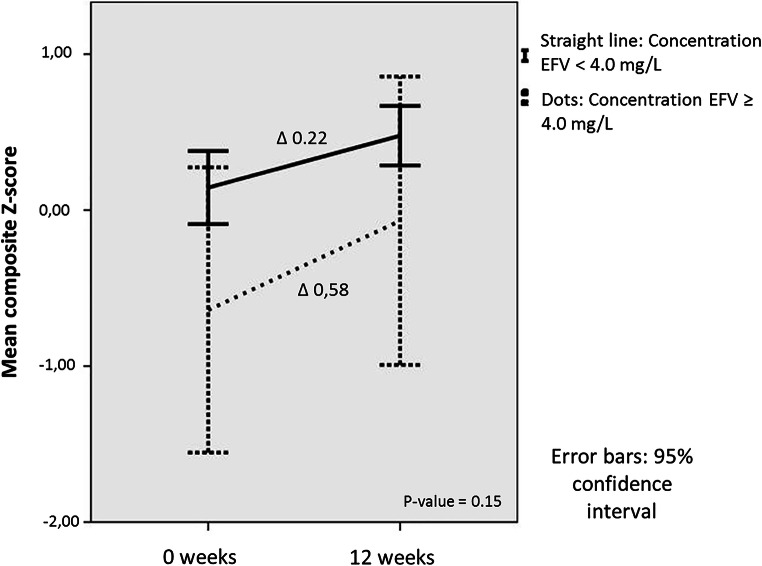
Mean composite Z-scores with confidence intervals in the switch group, divided at baseline in therapeutic and elevated concentration of efavirenz

Next, we evaluated only the group of participants that switched from a regimen with efavirenz to a regimen without efavirenz in order to study the effect of efavirenz discontinuation. Differences (‘delta-scores’) between NPA Z-scores on baseline and end of study were investigated. In the switch group, 5 participants had an elevated concentration, and 33 participants had a therapeutic concentration of efavirenz (Fig. 1). All participants improved on the second NPA due to a learning effect. Participants with an elevated concentration at baseline had a higher delta, i.e. improved more on composite Z-score (0.58; SD 0.32 versus 0.22; SD 0.54, *p* = 0.15) compared with those with a therapeutic concentration. When looking at subdomains, the group with an elevated baseline concentration of efavirenz improved more on the domains verbal (0.47 SD 0.42 versus 0.15 SD 0.64, *p* = 0.63), attention (0.98 SD 0.67 versus 0.46 SD 0.6 *p* = 0.33), speed (0.93; SD 0.73; versus 0.42; SD 0.41, *p* = 0.05), motor (0.80 SD 0.36 versus 0.54 SD 0.85, *p* = 0.18) and learning (0.80 SD 0.31 versus 0.41 SD 1.00 *p* = 0.65) (Fig. 2; Table 3). Although none of these improvements were statistically significant, a trend towards significance was seen in the domain speed.

**Table 3:** Difference (‘delta’) scores of NPA Z-scores in normal and high concentration of EFV groups within the switch group

Type of NPA Z-score	Concentration EFV < 4.0 mg/L	Concentration EFV ≥ 4.0 mg/L	*p* value
Composite median (IQR)	0.22 (0.54)	0.58 (0.32)	0.15
Domain verbal	0.15 ± 0.64	0.47 ± 0.42	0.63
Domain memory	− 0.24 ± 0.49	− 0.03 ± 0.73	0.48
Domain executive functioning	0.42 ± 0.52	0.33 ± 0.30	0.82
Domain attention	0.46 ± 0.60	0.98 ± 0.67	0.33
Domain speed	0.42 ± 0.41	0.93 ± 0.73	0.05
Domain motor	0.54 ± 0.85	0.80 ± 0.36	0.18
Domain learning	0.41 ± 1.00	0.80 ± 0.31	0.65

Values shown as mean ± SD, unless otherwise specified

*Difference between groups considered significant (*p* value < 0.05)

*NPA* neuropsychological assessment, *EFV* efavirenz, *IQR* interquartile range, *SD* standard deviation

Because of the significant difference between the two groups in employment status and the near-significant difference in nadir CD4, we ran an extra GLM including these factors, and they had no effect on both outcomes (Z-score or delta Z-score).

### NFL as a biomarker for neurocognitive impairment

To evaluate whether Nfl plasma concentration is related to neurocognitive impairment, a linear mixed model with random intercept was built. The full model contained 8 covariates (age, use of psychoactive comedication, duration of cART and HIV infection, current and nadir CD4, efavirenz concentration and time point in study (baseline or week 12)). A significant relation was found between composite Z-score and time point in the study indicating that all patients increased their Z-score on the second time point, which can be explained by a learning effect from doing the NPA for the second time. (coefficient = 0.26, standard error = 0.0746, *p* = < 0.01). No other factors had a significant association with composite Z-score (Table 4). A final model was created by using the backward method, containing four variables (use of psychoactive comedication, duration of cART, efavirenz concentration, time point in study). Plasma NFL still did not show a significant association with the outcome composite Z-score (coefficient = 0.0012, standard error = 0.0030, *p* = 0.71).

**Table 4:** Full linear mixed model on the outcome composite Z-score

Fixed effect	Coefficient	Standard error	*p* value
Intercept	0.38381	0.66321	0.57
Age, in years	− 0.00065	0.01631	0.97
Use of neuropsychiatric medication	0.15100	0.33070	0.65
cART, in months	− 0.00378	0.00441	0.40
CD4 count, in cell/mm^3^	− 0.00008	0.00030	0.78
CD4 nadir, in cell/mm^3^	− 0.00010	0.00072	0.89
HIV disease duration, in months	0.00006	0.00345	0.99
EFV concentration, in mg/L*	− 0.07816	0.05470	0.17
Plasma NFL, in pg/mL	0.00117	0.00355	0.75
Time point in study**	0.26045	0.07457	< 0.01

*Efavirenz concentration transformed to log variable

**Time is defined as a categorical variable

*cART* combination antiretroviral therapy, *HIV* human immunodeficiency virus, *NFL* neurofilament light

Next, we investigated which variables had an effect on plasma Nfl in order to identify possible disturbing influences in Nfl levels. Univariable linear regression on plasma neurofilament light at baseline showed no significant association, except for HIV disease duration (*p* = 0.32; *p* = 0.02) (supplemental data). For the multivariable linear regression, 5 variables were entered in the model, age (*p* = 0.12), months on cART (*p* = 0.08), CD4 nadir (*p* = 0.96), HIV disease duration (p = 0.02) and viral load at baseline (*p* = 0.14) (supplemental data). When creating a multivariable linear regression model using the backward method, only HIV disease duration remained statistically significant (coefficient 0.090, standard error = 0.037, *p* = 0.02).

## Discussion

This is the first study that aimed to investigate the effect of efavirenz exposure, measured by plasma concentration, on objectively measured cognitive functioning in cognitively asymptomatic people with HIV. An elevated efavirenz concentration was associated with worse cognitive functioning overall and in different domains (verbal, executive functioning, attention and speed). Furthermore, discontinuing efavirenz resulted in more neurocognitive improvement in those with an elevated efavirenz baseline concentration compared with those with a therapeutic efavirenz baseline concentration. However, this effect was not statistically significant, apart from a trend in the subdomain speed, most likely due to a limited sample size. Moreover, when exploring the use of plasma Nfl as a biomarker in neurocognitive functioning, no association between plasma Nfl and composite Z-score was found.

Patients usually switch to another (efavirenz-sparing) regimen when they experience neurocognitive side effects. However, there is a group of patients that tolerate efavirenz and do not experience a clinically significant effect on cognition. The strength of our study lies in the fact that we analysed the effect of efavirenz concentration in these cognitively asymptomatic people with HIV, as opposed to patients with overt neurocognitive complaints. The domains that showed the largest effect of elevated efavirenz concentration (speed and attention) are also the cognitive domains that were mostly affected by discontinuing efavirenz in the ESCAPE trial.

Although toxicity thresholds from 2.74 to 4.7 mg/L for efavirenz have been used in studies (Núñez et al. 2001; Gutierrez et al. 2005), we chose to use a cut-off level of 4.0 mg/L for efavirenz concentration, seeing as this is the most used threshold in the international literature (Marzolini et al. 2001; Gallego et al. 2004; Kappelhoff et al. 2005; Burger et al. 2006; Naidoo et al. 2014). In order to measure cognition in a fast and less time-consuming manner, there is a need for biomarkers that can preferably be measured in plasma. Plasma Nfl has been shown to be useful in providing an indication of active CNS injury in HIV infection (Gisslén et al. 2016; Anderson et al. 2018). However, these studies found the most significant results in patients with HIV dementia or in untreated people with HIV. The current study is the first study that aimed to explore the utility of plasma Nfl as a biomarker in treated cognitively asymptomatic patients with HIV. An association between plasma Nfl and composite Z-score was not found. Moreover, the concentration of efavirenz did not have a significant association in this relationship, and therefore, the switch in regime was not a contributing factor in explaining the association between plasma Nfl and composite Z-score in this analysis. The hypothesis of this study that efavirenz causes axonal damage, and therefore results in rising plasma Nfl levels, could therefore not be proven in this study. The negative effects of efavirenz on the brain might be explained by other mechanisms than axonal damage. In vitro studies did show a larger effect of efavirenz on dendrite cells than axonal cells (Robertson et al. 2012; Tovar-y-Romo et al. 2012; Ciavatta et al. 2017). Furthermore, most studies found that neuronal death, which would also be measured by Nfl, was not the major reason for neurotoxicity of efavirenz (Robertson et al. 2012). Another explanation could be that plasma Nfl is not as sensitive as CSF Nfl, as is suggested in animal studies (Bacioglu et al. 2016).

There are some limitations to this study. First, the small number of participants was due to the limited number included in the time-consuming ESCAPE trial. Since no power calculation was done for this sub-analysis, it is possible that the used numbers do not provide sufficient power for the analysis. Moreover, there was a substantial difference between the amount of patients in the two groups (6 versus 48) that further reduced our power. Second, in contrast to previous reports on Nfl (Gisslén et al. 2016), in this study, plasma Nfl did not correlate with age. This could be explained by the fact that the variation in age was limited in this population due to the study inclusion criteria. Moreover, studies have shown that the correlation between age and Nfl levels is more evident in healthy controls (Khalil et al. 2013). However, this study found a significant correlation with duration of HIV infection and plasma Nfl levels. This effect might be explained by the hypothesis that HIV infection itself together with the accompanying chronic immune activation has an added neurotoxic effect. Furthermore, people who have been infected with HIV for a longer period have consequently been on more and older cART regimens. Previous cART regimens possibly did cause axonal damage and therefore caused higher levels of plasma NFL (Schmued et al. 1996; Fodale et al. 2005; Pettersen et al. 2006; Xu et al. 2014). Considering the fact that detecting Nfl in plasma is a recent development, an all-encompassing answer explaining all possible factors influencing plasma/serum NfL levels is not yet available. Therefore, it is important to further investigate the applicability and validity of plasma Nfl as a biomarker, preferably in larger cohorts of people with HIV suffering from neurocognitive impairment.

In conclusion, elevated serum efavirenz concentration is associated with worse cognitive functioning, and there are signs that subsequent discontinuation results in improvement of cognitive functioning compared with those with normal concentrations. Plasma Nfl is not suitable as a biomarker for cognitive damage in this group.

## Electronic supplementary material

ESM 1 (DOCX 13 kb)
