# Supplementary material for: High efavirenz levels but not neurofilament light plasma levels are associated with poor neurocognitive functioning in asymptomatic HIV patients
Source: J Neurovirol. 2020 Jun 10;26(4):572–80. doi: 10.1007/s13365-020-00860-1 (PMC7438296; doi:10.1007/s13365-020-00860-1)
Supplement: Supplementary file 1 — (DOCX 13 kb) [file 13365_2020_860_MOESM1_ESM.docx]

**Supplemental Data**

**Table 1*.*Univariable linear regression on plasma neurofilament light at baseline**

| Variable | Pearson correlation (ρ) | p-value |
| --- | --- | --- |
| Age, in years | 0.21 | 0.12 |
| Use of neuropsychiatric medication | 0.19 | 0.17 |
| cART, in months | 0.25 | 0.08 |
| EFV, in months | 0.13 | 0.38 |
| CD4 count, in cell/mm3 | -0.11 | 0.46 |
| CD4 nadir, in cell/mm3 | -0.01 | 0.96 |
| HIV disease duration, in months | 0.32 | 0.02 |
| Concentration of EFV, in mg/L | 0.08 | 0.59 |
| Viral load, in copies/mL | -0.21 | 0.14 |

*Abbreviations: cART = combination antiretroviral therapy; EFV = efavirenz; HIV = Human immunodeficiency virus*
